# Depression, Deprivation, and Dysbiosis: Polyiatrogenesis in Multiple Chronic Illnesses

**DOI:** 10.1007/s11013-020-09699-x

**Published:** 2021-02-06

**Authors:** Stefan Ecks

**Affiliations:** Social Anthropology, University of Edinburgh, Chrystal Macmillan Building, George Square, Edinburgh, EH8 9LD UK

## Abstract

Biomedicine tends to treat “mental” illnesses as if they could be isolated from multiple social and somatic problems. Yet mental suffering is inseparable from complex somatosocial relations. Clinical fieldwork in a deprived area of the UK shows that nearly all the people treated for “depression” are chronically multimorbid, both in their bodies and in their social relations. Mental suffering is co-produced by poverty, trauma, and excessive medication use. Patients’ guts are as imbalanced as their moods. Single vertical treatments make them worse rather than better. In the UK, patients in poorer neighbourhoods do not “lack access” to healthcare. If anything, they suffer from taking too many medications with too little integration. I conceptualize the bad effects of excessive interventions in patients with multiple chronic problems as polyiatrogenesis.

## Mental Health Beyond the Brain

Can “mental” health be distinguished from “health” in all its physical and social complexities? Where does “mental” health reside, is it “within” the body but somewhere apart from bodily health? Is it also located outside of the body, in cultural contexts and social ties? The links between mental and somatosocial health—if they even are “links” between separate elements—are hard to capture. 20th-century psychiatry made a “metaphysical wager … that madness had its roots in the body” (Scull 2015:1067), yet how exactly mental, physical, and social health are connected remains blurry.

Public and global health approaches state that mental health cannot be separated from overall health, yet which comes first, whole health or mental health? The motto of global mental health is “no health without mental health” (Prince et al. 2007). All dimensions of life are enhanced when “mental” health is improved. Better mental health could reduce all physical disorders (including infectious diseases) and all social problems (including poverty). Mental health should be given primacy in “all aspects of health and social policy, health-system planning, and delivery of primary and secondary general health care” (Prince et al. 2007:859). But somatosocial interventions might be just as important for mental health as mental health interventions. If mental health cannot be separated from bodily and social health, why is it that both research and clinical practice still treat mental health in isolation (Garcia 2010:215)? Why do the psysciences struggle to conceptualize mental health beyond the brain? There are ideas about a “convergent” approach to mental, somatic and social health (Patel et al. 2018:18), but it is still in its beginnings.

It took psychiatrists until the 1970s just to acknowledge “comorbidity,” defined as the coexistence of an “index” disorder and a “secondary” disorder (Feinstein 1970; Weaver, Barrett, and Nichter 2016). Even then, the comorbidities described were other mental problems, for example, depression coupled with anxiety, or depression with somatized pain (Kleinman 1977; Lilienfeld and Treadway 2016; Lupien et al. 2017). Mental/physical comorbidities, such as depression with diabetes, were only examined since the 1980s. The biomedical demand for diagnostic specificity continues to hinder a comprehensive understanding of mental, physical, and social health in all its complexity.

While “comorbidity” research only emerged in the 1970s, “multimorbidity” research is even more recent. Multimorbidity occurs when the same person suffers from two or more chronic disorders. The disorders can be mental, noncommunicable, or infectious (Academy of Medical Sciences 2018:6). A serious attempt to study multimorbidity only came about when the extent and severity of multiple chronic disorders could not be overlooked any longer (Busija et al. 2019:1). Until the 2010s, multimorbidity did not appear as a separate category because of biomedicine’s “vertical” attention to specific diseases and the neglect of “horizontal” linkages (Whitty et al. 2020). Biomedicine looks for specific aetiology and specific treatment. The “medical model” searches for specific causes of disorders and tries to find therapies that target specific causes (White 2006:141–142). But specificity is hard to find within multimorbidity.

Multimorbidity is increasing globally. In high-income countries, multimorbidity accounts for up to half of the overall disease burden (Garin et al. 2016; van der Aa et al. 2017), for half of all primary care consultations, and for at least 70% of healthcare expenditures (Aiden 2018; Kaufman 2015). One reason why multimorbidity is increasing is because life expectancy is increasing: the older the person, the more likely the co-occurrence of three or more chronic problems. The majority of people over 65 are multimorbid. Longer lifespans are not the only reason for rising rates of multimorbidity. Even where life expectancy stagnates, multimorbidity keeps rising. Many factors other than life expectancy co-produce multimorbidity, but these remain ill-understood.

Biomedicine’s drive to find the “best evidence” turned to ever greater specificity (Adams 2002, 2016). The pinnacle of best evidence, the randomized controlled trial, is predicated on specificity (Timmermans and Berg 2010). RCTs are *designed* to exclude participants who are multimorbid and who are on several chronic medications because they mess up results (Upshur 2005). That is why people older than 70 are routinely excluded from trials (Onder 2013; Kaufman 2015). RCTs are not well suited to understand several co-occurring problems, be they mental or physical. Attempts have been made to use RCT designs to study mental health with physical comorbidities in a context of social deprivation, but RCTs miss too many dimensions by trying to control all variables (Coventry et al. 2015). Making RCTs the gold standard of evidence creates a system that “focuses on translating partial statistical lives of patients with single diseases into the real lives of patients with multimorbidity, with a systematic blindness to how harmful this is” (Mangin and Garfinkel 2019:1).

Even when multimorbidity is recognized by clinicians, they struggle to deal with it. Clinical guidelines follow the single-disease paradigm (Guthrie et al. 2012). Physicians have to treat problems in isolation from each other. The more a health system is compartmentalized, the more likely it is that multimorbidity goes unrecognized. Patients with multiple chronic problems experience biomedical care as lacking coherence (Schiøtz, Høst, and Frølich 2016).

Relative social inequality co-produces multimorbidity. The most-cited study on multimorbidity is by Barnett and colleagues (2012), who analysed 1.8 million Scottish patient records. Already a decade ago, a quarter of all Scots were multimorbid. A third of these multimorbid patients had both mental and physical problems. Socioeconomic deprivation produces multimorbidity at a much younger age: “young and middle-aged adults living in the most deprived areas had rates of multimorbidity equivalent to those aged 10–15 older in the most affluent areas” (2012:39). In deprived neighbourhoods, people in their 40s and 50s are as likely to have multiple chronic problems as 60- or 70-year-olds in the average population. In the Barnett study, the link between multimorbidity and socioeconomic deprivation is strong in mental problems. 19.5% of people in affluent areas are multimorbid, compared to 24.1% in deprived areas (a ratio of 1:1.2). In turn, 5.9% of people in affluent areas have mental comorbidities, compared to 11% in deprived areas: a ratio of 1:1.8 (Barnett et al. 2012). That means that socioeconomic deprivation affects mental health comorbidities significantly more than multimorbidity without mental problems. Depression clustered with chronic physical problems and social deprivation is associated with “the greatest decrements in quality of life” (Coventry et al. 2015:2). The cost implications are huge: when a patient with chronic physical problems also suffers from depression, the cost of care per patient increases by 45% (Naylor et al. 2012).

The cultural anthropology of health and illness could be expected to have a long tradition of studying multimorbidity. A holistic approach is constitutive of the field (Baer et al. 2012). Anthropologists have long been critical of biomedical specificity. They emphasize that health and illness can only be studied in cultural, economic, and ecological contexts. Finding a single cause for a single disease seems futile when there are so many cultural, social, economic, and ecological dimensions (Dubos 1987:102). Just like biomedicine prefers disease specificity, anthropology also finds it easier to work on single illnesses. Anthropology’s holism puts health problems into cultural contexts, but usually stays with specific illnesses (Browner 1999).

The most sustained anthropological work on multiple chronic problems is on syndemics, a term coined by critical medical anthropologists (Singer and Snipes 1992; Weaver, Barrett, and Nichter 2016). Syndemics are “aggregation[s] of two or more diseases or other health conditions in a population in which there is some level of deleterious biological or behaviour interface that exacerbates the negative health effects of any or all of the diseases involved” (Singer et al. 2017:941). The syndemics approach wants to “move beyond common medical conceptualisations of comorbidity and multimorbidity” (ibid.) by emphasizing structural violence (Farmer 1996) and social suffering (Kleinman, Das and Lock 1997). Poverty and trauma are seen as key risk factors. Biomedical “multimorbidity” does not make claims about social aetiology. By contrast, syndemics research always searches for underlying social suffering.

Syndemics research looks for systematic clusters of disorders, not just for heaps of separate problems. Several constellations that contain at least one mental health problem have been identified. Stall and colleagues (2003) found a syndemic of depression, drug use, intimate partner violence, and childhood sexual abuse among urban men who have sex with men. Mendenhall (2012, 2016, 2019) described a syndemic of depression, violence, immigration, diabetes, and abuse among Mexican immigrant women living in the USA. Syndemics “confuse the diagnostic process” (Singer et al. 2017:945) by blurring the distinct identity of “mental” health problems into all kinds of other problems.

As I show with clinical case studies from a deprived area in the UK, mental health problems regularly co-occur with somatic and social multimorbidities. In the clinic, no patient would had only one illness. Every patient had at least two chronic problems, most had five or more. The majority had mental problems along with physical problems. Among many physical complaints, metabolic and digestive disorders were always present. Depression and anxiety were the most common mental illnesses. Both mental and physical problems were often related to traumatic life events. Many traumatic events happened in relation to family members. Domestic violence and emotional abuse in intimate partnerships were common. Haunting memories of losses suffered were a recurrent theme. Among the many problems that the patients faced, there was no telling what a “primary” as opposed to a “secondary” problem might be.

As I will further argue, too many patients with multiple chronic health conditions do not improve with biomedical treatment. Socioeconomic deprivation deepens chronicity and can make problems almost intractable, but the treatments often appear to harm as well. I conceptualize the deepening of multimorbidity by multiple isolated interventions as *polyiatrogenesis—*medical harm caused by medical treatments on multiple fronts simultaneously and in conjunction with one another.

That psychopharmaceuticals can have many serious side effects is well known (e.g. Whitaker 2010; Healy 2012). Since the 2000s, pharmaceutical companies lost many multi-million dollar lawsuits for downplaying the heightened risk of suicide after starting on antidepressants, and these risks remain controversial today (Witt-Doerring and Mathew 2019). However, the bad effects of specific drugs are only the tip of the iatrogenic iceberg. Much more serious is the epidemic of iatrogenesis that is co-produced and deepened by the epidemic rise of overtreated multimorbidity, with or without psychopharmaceuticals as part of the mix. Multiple chronic problems create a situation where even drug “side” effects become indistinguishable from “primary” effects (see Etkin 1992; Landecker 2016; Hardon and Sanabria 2017).

Iatrogenesis can occur (1) when too many different treatments are given at the same time (polypharmacy); (2) when two or more drugs together produce adverse side effects (drug–drug interactions); and (3) when specific treatments harm (irrational or inappropriate treatments) (Novaes et al. 2017). Critical pharmacologists are observing a global “iatrogenic epidemic” (Mangin and Garfinkel 2019:1), which is linked to multiple medication uses. Some of these iatrogenic effects are known, for example, beta-blockers prescribed for hypertension can worsen asthma and mask dangerously low blood sugar levels in diabetics (Onder 2013). The more disorders and their treatments multiply, the less possible it is to control for harmful effects.

When physicians keep firing several tablets at multiple chronic problems simultaneously, they can cause more harm than good. Lenore Manderson and Ayo Wahlberg point out that the “cascading of multiple medical conditions complicate treatment and its coordination” (2020:4). The reverse is even worse: the most harm ensues when the cascading of multiple treatments produces and aggravates multiple medical conditions.

When “mental” problems are inseparable from physical and socioeconomic difficulties, pharmacological targeting within a single-disease framework cannot succeed. In multimorbid patients, mental symptoms cannot be singled out for specific treatment. In turn, continued attempts at targeted treatments backfire. As the following case studies show, biopsychosocial multimorbidity gets exacerbated by the polyiatrogenic effects of uncoordinated treatments.

## Multimorbidity and Polyiatrogenesis: Clinical Case Studies

Fieldwork in a British care centre shows how mental suffering is embedded in multimorbidity, and how polyiatrogenesis deepens chronicity. The research was carried out in “Greyfield,” which is classified as one of the UK’s “most deprived” areas. The census classifies the local population as 90% “white British.” Most of the housing is residential. Greyfield was built in the 1960s and 1970s in the form of tower blocks and low-rises. It saw infrastructural improvements in the 1990s and 2000s but still has abject scores for health, income, employment, education, and crime. Greyfield’s average life expectancy is only 61 years, which is 20 years less than the UK average. Zimbabwe has a higher life expectancy than Greyfield.

Despite relative deprivation, Greyfield has no shortage of biomedical services. One of the infrastructural improvements since the 2000s was the construction of Greyfield Care Centre (GCC), which aims to integrate various medical and social care services. Alongside primary care, cancer support, dentistry, and midwifery, GCC also houses a clinic for Lifestyle, Nutrition, and Complementary Therapy (LNC). A decade ago, multimorbidity researchers advocated that clinical practice should move from a single-disease framework to complementarity: “A complementary strategy is needed, supporting generalist clinicians to provide personalised, comprehensive continuity of care, especially in socioeconomically deprived areas” (Barnett et al. 2012:37; see Mercer et al. 2012; Coventry et al. 2015). GCC moved towards complementarity but did not specify pathways and referral protocols. If and where patients are referred is hit and miss. The hegemony of biomedical doctors over GCC’s complementary providers is never questioned.

My fieldwork was conducted in the LNC section of GCC. I evaluated 80 case files, discussed 30 selected cases with therapists, and shadowed 40 consultations. The typical LNC patient was a white British woman in her 30s, not employed, and referred by a GP. Three quarters of patients were in their 30s and 40s. More than 80% were women. Only one third of clients had employment, the other two thirds were either on long-term sick leave, on unemployment subsidies, or they were retired. The largest number of patients came by referral from GPs. When making their referrals, physicians would usually write down one health problem as a reason for why they referred. They rarely indicated two reasons, hardly ever three, and never more than three. But when I began to work through the case notes and to shadow the therapists, it was clear that none of the patients fit into the straightjacket of a single-disease classification. The case studies highlight how “mental” problems are indistinguishable from bodily and social health, and how targeted treatment risks harming patients with through polyiatrogenesis.

### I’ve Been Suffering for So Long (Mary, 58)

Mary was a Greyfield resident in her late 50s. She had many children and grandchildren. Caring for family members consumed her entire day. Mary was chronically depressed. She had been on amitriptyline (Elavil) for longer than she could remember. Amitriptyline is an old generation tricyclic antidepressant. Due to its toxicity in chronic use, amitriptyline is not used as a first-line treatment. Common side effects include constipation. Amitriptyline is often prescribed for depression with chronic comorbid pain. Mary also suffered from irritable bowel syndrome (IBS), which made her abdomen swollen and painful. Amitriptyline was meant to suppress abdominal pain but it also worsened the constipation. Mary also had chronic sleep disturbance, chronic obstructive pulmonary disease (COPD), loss of feeling in her legs, recurring urinary tract infections, and chronic back pain. Mary was overweight and a smoker. Amitriptyline was just one among many medications: Mary also took laxatives, gabapentin and dihydrocodeine for pain, two different inhalers for COPD, and statins to lower blood pressure. Years earlier, a GP suspected an *H pylori* infection of her stomach. She was put on antibiotics, first for half a year, then continuously for another five years. The LNC practitioner, Iris, thought this was a “ridiculous response” by the GP. Many people had *H pylori* in their stomach without any symptoms, “what matters is how your body responds to that.” The antibiotics knocked out Mary’s “healthy gut bacteria.” Iris thought that Mary’s metabolism was wrecked by severe dysbiosis, that is, a preponderance of harmful microorganism in the digestive tract.

A few weeks before her first LNC consultation, one of Mary’s relatives was murdered, but she mentioned this only in passing. Mary described many other tensions in the family. Mary did not like to drink water and instead “lives on coffee.” Most foods made her gag with disgust. Iris suspected an intolerance to wheat and dairy, and suggested that Mary should have her coffee without milk. Iris selected a set of natural remedies to rebalance the gut flora. Mary’s diet was very low in fibre, but Iris thought that it would be difficult to change her food routines when everyone else in her family continued as before. Iris said she feels “incredibly self-conscious” as a “middle class person” to tell Mary that she should radically change her lifestyle. Iris also said she was “shocked” about how little people got told by doctors about diet, gut health, and medication side effects: “no one bothers to speak to them.”

Over several months of treatment, Mary’s overall health improved a little bit. Mary enjoyed being listened to: “It’s like counselling here,” she said. The LNC treatments were helping her. She still had sleep problems, going to bed at 9pm, then waking up at 2am when she would “wander about the house” and feel tired for the rest of the day. Mary still felt low in energy and did not want to eat. She only had one bowel movement every four days. Eating a few raisins and nuts seemed to help with the constipation. Iris asked if Mary ate more vegetables to “add bulk to your stool,” and Mary said she did not like them, but forced herself to put them into soup. Tomatoes appeared to be a problem. The other day she put brown sauce on a sandwich and felt horrible. “What the hell is going on?,” she thought, but then she realized that brown sauce is made from tomatoes.

Mary summed up her situation like this: “I’ve been suffering for so long, and all that happened was that I was given this tablet or that pill.” No one sat down with her to explain the treatments or why she was given the antibiotics for such a long time. “I never had a follow-up” on whether she still “had the virus” (meaning the *H pylori* bacteria). Iris explained that the presence or absence of *H pylori* would only be one piece of the puzzle, and that taking antibiotics for so long would certainly have a bad effect on the gut. The medications had caused “dysbiosis.” Iris recommended that Mary take psyllium with her food to make it easier for her to pass stool more regularly. Reflecting on which of the diet changes worked, Mary said she tried the tea that Iris had recommended earlier, but she found it disgusting and stopped taking it.

### They’re Just Firing Tablets at Me (Molly, 37)

Molly was a 37-year-old woman from Greyfield. When she first came to LNC, she was on citalopram (Celexa), an SSRI commonly prescribed for depression. She had also been on propanonol, a beta-blocker, for many years. Both citalopram and propanonol were meant to help her against anxiety. The anxiety came from many sources, one of which was her fear of having diarrhoea when she was out of the house. Molly found her job as a care worker very stressful, especially when she was working late hours. Her bowel movements caused pain and anxiety. Any kind of “stress” got to her stomach.

The first time she was diagnosed with IBS was when she was 11 years old. She got some treatment and the IBS subsided. Then, five years ago, Molly had a protracted breakup from her husband, during which time she and her son suffered “domestic abuse.” That led, in turn, to lots of “behavioural problems” in her son. When asked by the LNC therapist, Heather, if she had time to look after herself, Molly said that “my self-care is really bad, I know that it is really bad.” She was aware of various food triggers that she was trying to avoid. A few weeks earlier she had a spicy curry that caused pain and diarrhoea.

Molly had a prescription for hyoscine butylbromide (Buscopan), a medication for abdominal cramping. Molly also took a dose of imodium every morning and had become completely dependent on it. Imodium (loperamide) is an over-the-counter treatment for acute diarrhoea. The medication slows down digestion, and should not be used for more than 48 hours. Molly complained about her daily eating habits, especially too many potato chips: ”Pringles are my downfall.” Molly drank three to four litres of Coca-Cola throughout the day: ”I am really bad with fizzy drinks.” Molly’s weight was 20 stone. At her body height, her BMI was 45, putting her into the obese category. She said she had always been “big.”

Molly was diagnosed with type II diabetes a few months prior to coming to LNC. Heather asked Molly if a GP had explained the links between diabetes, blood glucose, and high-sugar drinks. Molly said the doctors did not have time to explain what she should eat. Instead, “they’re just firing tablets at me.” She had not been told by anyone face-to-face how to manage her diabetes. Asked about her blood sugar readings, Molly replied that no one had done a blood test on her and she did not know about it. The diabetes had been detected by an optician when she took an eye test for new glasses, not by a GP. Heather applied a blood sugar test and found a glucose level of 17.9 mmol/L. This is so high as to be life-threatening.

Given the worrying blood sugar levels, Heather wanted to know if Molly was taking any medications for her diabetes. Molly pulled out a black rubbish bag: “my lucky bag of medicines,” she laughed. Molly said that she is taking two medformin for diabetes every morning. Heather told her that she should find out how to monitor her blood sugar, not least because her levels are so high *despite* already taking medformin.

Molly kept unpacking her “lucky bag” and put more drugs on the table: citalopram; propanonol; Fianola, a contraceptive; an antihistamine for allergies; omeprazole against gastric reflux; gentamicin, an antibiotic drug; and a high dose of ibuprofen. Molly continued to unpack various nonbiomedical remedies, including sage pills and matcha green tea pills, which were meant to help against “hot flashes.” She said that she is a “hot person,” “burning up very quickly.” She joked that she should “move to Iceland” to feel more comfortable.

Molly’s initial reason to come to LNC was a growth in her foot. A GP had told her it was a “ganglion.” It caused her much pain and restricted her movement. By overcompensating with her other foot, she developed plantar fasciitis and dislocated a bone. She has been on the phone with the GP about the ganglion but was told that there was nothing else they could do. Heather probed what the pain in her foot felt like and Molly told her it was like “pins and needles,” which Heather said might come from the diabetes. Asked about the GPs’ treatment for plantar fasciitis, Molly said that she has not been told what she could do about it. Heather explained what plantar fasciitis is and showed her a few stretches to alleviate the pain.

At the close of the consultation, Molly was told to come back soon for a fuller “systems review.” The therapist advised that Molly should stop drinking fizzy drinks immediately and keep an eye on her blood sugar levels. Molly asked if juices or fizzy water would be alright to have instead, but Heather told her that neither of them would be good for her, both because of the sugar and because of the fizz. Molly thanked Heather for taking so much time: GPs only ever spoke to patients briefly, and only about a specific ailment, she said.

### Now I’m Probably the Worst I’ve Ever Been (Rob, 38)

Rob was a Greyfield man in his late 30s. He came to the clinic for depression, anxiety, panic attacks, headaches, and sleep disturbances. He also suffered from chronic back pain, which two previous surgeries could not get rid of. Rob had been on different SSRI antidepressants over the years. He continued taking the medications but he was not convinced they were helping him in any way. “Now I’m probably the worst I’ve ever been. Other people have said that as well.” Asked if he was feeling suicidal, Rob said that he had often thought about killing himself, but more in the past than currently. He was not feeling suicidal presently. His mood was going up and down, cycling through “two good days, two bad days.” A “bad day” is when he stayed in bed all day with the curtains closed. He had come to LNC because he wanted to get off the antidepressants, they seemed to give him no relief, in fact, they were making him worse. He had tried to replace the antidepressants with herbal remedies: “I tried St. John’s Wort and I felt better. Certainly at the time when I took it, it felt better.” Rob had also been prescribed counselling. Initially he had enjoyed the counselling sessions, which used cognitive behavioural therapy: “I had a really cool counsellor, I really liked her.” Then the service assigned a different counsellor with whom he did not get along, so he stopped going.

Rob traced his depression and anxiety back to his childhood: as long as he could remember, he was disgusted by meat: “I don’t have any views or opinions about it, I just don’t like meat.” His parents had no understanding for this and force fed meat to him. Since then he had “a fear of foods.” His job as a self-employed car recovery mechanic meant that he worked irregular hours outside the house. When he is out, he does not eat: “I cannot find the foods that I like in the shops. That affects my mental health and my mood.” His fear of foods also caught up with him in his leisure time. Recently friends prepared a meal. They assured him that there was no meat in it, but he still felt extremely anxious of eating anything in the company of others and not knowing what was in the food: “I got a panic attack.” Asked if he had any “safe foods,” Rob said that he can usually have plain pasta with a bit of olive oil. Rice and some vegetables were also fine. He had recently discovered “vegan superfood supplements,” which he added to some of the meals.

Rob also suffered from acid reflux, which, he said, was linked to all the cannabis he was smoking. He went through an ounce of weed per week. He took omeprazole to counter the reflux. He was constantly experimenting with different kinds of cannabis in different dosages at different times of the day to figure out which produced the least reflux: “I’m trying to understand the differences.” He found that too much tobacco in the joint increased reflux. Some strains of cannabis were better than others. However, each strain also had other effects on moods and sleep. Rob had severe problems falling asleep at night, “my mind is racing.” He was always “thinking, totally overthinking.” He had heart palpitations because of his anxious thoughts. He would go to bed at 3am and wake up at 10am with a headache. He said that he wanted to face his fears: “I don’t want to deflect anything from my life.” The LNC therapist, Violet, prescribed a mixture of natural remedies, including rosemary and camomile extracts, which would make him feel more energetic and lift his mood. Violet also recommended that Rob works towards a more regular eating schedule and that he adds more healthy fats to his diet.

### I’m Not Suicidal, Just So Fed Up (Sarah, 45)

Sarah was a Greyfield woman in her mid-40s. She suffered from depression, anxiety, and posttraumatic stress. Sarah’s poor mental health was inseparably linked to her physical suffering. She has had inflammatory bowel disease since she was 14 years old. Bouts of diarrhoea were frequent. Her mother’s death deepened her gut problems and she developed ulcerative colitis. The pain and the nutrient deficiency greatly hampered her immune system.

Five years prior to coming to LNC, she had undergone surgery to remove parts of her colon to counter the pain of the colitis. In the course of the colon operation, the surgeon performed a full hysterectomy without taking Sarah’s consent. Sarah was only told about the hysterectomy when she woke up from the anaesthesia. She felt utterly violated by what the surgeon had done to her. In his defence, the surgeon shrugged and said that “the uterus was in the way.”

As it happened, none of the surgeries took the pain away. Instead, Sarah suffered additional nerve damage that radiated through her body, even her feet were either feeling numb or “burning like fireworks.” Feeling numb was still better than feeling the searing pain, she said. Sarah was also continued on several antibiotics because her gut remained “infected” even after the surgery. The main benefit of the surgery was that she passed stool and urine less frequently than before, going from “three times in 15 minutes” to “ten times in 24 hours.” Sarah slept poorly since she was 14 years old, both for the pain and for the frequent urge to go to the toilet. Recently she had been given treatment for sleep apnoea, which consisted in her putting on a breathing mask during the night. She had strong night sweats.

The hysterectomy pushed Sarah into a sudden and crippling menopause, with strong hot flashes and severe hormonal disturbances. In one of the LNC consultations, Sarah talked about a meeting she has had with the surgeon who did the hysterectomy. She said that they “were very polite to each other” but that she left disappointed. Sarah hoped the surgeon would apologize for the harm he had done to her, but she neither received an apology nor any kind of recognition. The surgeon discharged her as fully healed, despite the fact that the pain continued and her GP put her on more antibiotics. Her digestion continued to be extremely poor, with a lot of undigested foods and mucus in her stool. Still, she said she managed to go out of the house. “I’m proud of myself,” she said, with tears in her eyes.

On another visit to LNC half a year later, Sarah told the therapist that the prolonged treatment with antibiotics did not make her better, in fact her digestion was now worse than ever before. She said that her gut had been completely colonized by “bad bacteria.” She had some growth in her nose that blocked her breathing and gave her a thumping headache. Her immune system was weakened and she was prone to coughs and colds. Her energy levels were lower than before. Sarah said she could not manage daily chores any longer. She was just lying in bed for hours, “listening to the world.” Her husband said she should get up and move but she could not get herself motivated. She had said to her husband that she wanted a gun to shoot herself, but it was only meant as a joke. “I shouldn’t have said that,” Sarah laughed, “I’m not suicidal, just so fed up.”

## Cultures Out of Balance

Complementary therapies are said to work more holistically than biomedicine because they want to understand the root causes of illnesses and to use therapies for long-term, sustainable improvement (Maizes, Rakel, and Niemiec 2009). In my interviews with Greyfield’s LNC practitioners, they disavowed the idea of “holistic” therapy because they feel that they could not deal with health in all dimensions. But what they could do, they said, was to take a more comprehensive view of patients’ complicated lives and help to rebalance their guts. An acute awareness of the limits of what patients can change in their lives ran through all the consultations. LNC therapists strain to never criticize patients for not sticking to a prescribed regimen: “They got so much else going on in their lives,” Jasmine said. Greyfielders suffer from “recursive cascades” (Manderson and Warren 2016), where one problem feeds into another. Patients should never be judged for what they cannot do, the therapists pointed out. Many patients were so “dissociated” from their own bodies that they could not feel what was happening to them. LNC practitioners said that Greyfield patients have very “complex” presentations, and the more complex, the less “confident” they could be about therapy. If the problem is simple, the remedies can also be simple. More complexity means less confidence, and less confidence means that they have to try many different routes. LNC practitioners thought that patients’ bodies and lives are so complicated that truly holistic therapy becomes impossible. The GCC patients had signs of long-term polyiatrogenesis, making their conditions seem intractable.

LNC practitioners were mindful of the limits of their therapies. From among the 30 case files that I discussed with them, they only identified one where they felt that the therapy gave the client significant relief. In Greyfield, therapeutic success could only be measured by tiny, incremental improvements (Csordas 2002). Still, LNC gave patients a sorely needed “space to reflect” (Iris). An open-ended assessment of illness histories, food habits, and various stresses allowed people to have “new realizations.” Giving patients time to speak openly and open endedly is the basis of their sympathetic care (Cubellis 2020).

LNC consultations start with one-hour assessments of patients’ medical and personal histories. The therapists then put together a treatment plan tailored to the individual patient. The LNC approach includes the use of plant-based remedies deemed to have no harmful side effects. Remedies are always combined with advice on diet and on daily habits. Food advice is based on a detailed discussion about everyday routines, noticeable intolerances and dislikes, as well as food triggers. LNC practitioners ask about typical days of eating. Some patients are asked to keep a food diary. Whenever therapists suspected food allergies or food triggers, they recommended an exclusion diet to tease out the offending items.

The diets and remedies prescribed in LNC “speed up” metabolism, “tone” porous membranes, or “calm” the nervous system. The therapists said that there could never be a “perfect prescription”: a complementary approach was always about experimentation and constant adjustments. The effects of the remedies varied by patients’ individual constitutions. Biomedical practitioners used fixed algorithms: if X disease is present, then Y drug should be given. “They have flowcharts” (Violet). But in complementary therapy, one-size-fits-all treatments could never work.

As the treatments unfold, LNC therapists ask clients how they experience the remedies and diet recommendations. The taste of some remedies could be unpleasantly bitter or astringent, which made clients avoid them. If a patient strongly dislikes a remedy or experiences adverse effects, the practitioners change the kinds, combinations, or dosages.

Complementary therapy is limited by clients’ “complex” constitutions and life circumstances (Ecks 2013:102, 137). In the case discussions, the LNC therapists explained that treatments can either be slow and gentle, or quick and “heroic.” Heroic prescribing uses substances that trigger a strong response in the body. “Speeding up the liver” releases toxins, resulting in an initially negative reaction like a skin rash. The skin is an “eliminatory organ” and an initial skin inflammation can be a good sign. Other heroic treatments can affect hormones or increase blood circulation, which helps in ridding the body of toxins. This is the general theory, but it could not be applied in Greyfield. Local patients’ bodies were so weakened and so complicated that heroic treatments were not recommended. Greyfield clients “need time to readjust,” they should “not be pushed too hard.” Patients resisted strong treatments, finding them far too taxing. In some of the case files analysed, LNC therapists commented that the body is made up of different layers, and “emotions are the deepest layer.” Heroic treatments could release not just too many toxins at once, but also too many negative emotions at once. The therapists highlighted how complex the body is, and how unpredictable responses can be. Why some therapies had certain effects in some patients and not in others was often unclear, they said.

Much relief could come from enhancing a patient’s microbiome. Links between illness and the microbiome, as the aggregate of microbiota that live on or within human tissues, has gained huge interest over the past decade (e.g. Turnbaugh et al. 2007; Perro and Adams 2017; Wolf-Meyer 2017; Lock and Nguyen 2018). The connections between the microbiome, inflammation, and mental health are slowly being recognized in mental health research as well (Patel et al. 2018:16). In LNC, the microbiome is a key concept. “Dysbiosis,” as microbiota cultures out of balance, was a term the therapists used often. Fruits, vegetables, and plant-based remedies could restore balance and enhance microbiome diversity. Working with the microbiome meant treating patients from inside out, instead of suppressing outward symptoms.

The microbiome could be influenced in several ways. First, some vegetables could “feed” the microbiome and make it more diverse (e.g, garlic, onions). Second, pathogenic microorganisms could be minimized by foods with antimicrobial properties (e.g. turmeric, garlic, cinnamon, ginger). Many chronic ailments caused tissue inflammation. Treating the microbiome reduced inflammation, and reducing inflammation improved overall health. LNC practitioners also mentioned the microbiome to patients, explaining briefly what it is and how treatment is connected to it. Microbiome therapy includes paying attention to bloating, nausea, acidity, and reflux.

LNC practitioners said that it is far more difficult to make patients change their diets than to make them take remedies. Even asking patients about what they are eating was difficult: “I *hate* talking about patients’ diets. I don’t handle food histories very well (laughs)” (Heather). There were many reasons why talking about diet was so difficult. Patients’ recall of what they were actually consuming was unreliable. Even more difficult is talking about diet without coming across as judgmental. Food was a very sensitive topic: “I feel like food is such an incredibly complex subject. It has all these social and emotional connotations. And I feel very aware of class. I don’t ever want to appear like lecturing someone, or appear judgmental about how they eat” (Violet). Social class strongly influenced if people can change their diet or lifestyles. Changing clients’ diets was hard because Greyfield is a “food desert” with limited choice, and many people could not afford fresh fruit and vegetables. Questions about food should never be asked with an assumption that a bad diet is the patients’ individual fault, or that change just required a bit more “education.” The LNC patients were not suffering because they “lacked compliance” with prescribed treatments (Whitmarsh 2013; Wolf-Meyer and Callahan-Kapoor 2017). At the same time, LNC practitioners wanted to avoid the “arrogant” view that patients are so stuck in their miserable lives that they are unable to make any changes. Either way, dietary change was extremely hard to get right with Greyfielders.

## The Harms of Fragmentation

Greyfield patients show many signs of polyiatrogenesis. Most have protracted digestive problems that get worse through chronic use of antibiotics and painkillers. Digestive disturbances caused by pharmaceuticals trigger cascades of other symptoms that provoke more interventions. The treatments that Greyfielders receive often cause more damage than do good. Many physicians acknowledge that patients with multimorbidities experience care as fragmented (van der Aa et al. 2017; Penm et al. 2017). Physicians tend to tackle separate disorders rather than a patients’ whole health. Time and resource limitations make it hard for them to explore how distinct disorders might be linked horizontally (Mercer et al. 2012; Whitty et al. 2020). When a patient moves from primary to tertiary care, the fragmentation gets worse. Even in systems that prioritize primary care, it is difficult for any individual physician to see the effects of isolated treatments. Multiple treatments worsen an “invisible epidemic” of iatrogenesis (Mangin and Garfinkel 2019). Molly’s “lucky bag” or Mary’s complaint that doctors are just firing tablets are idioms of distress about the deepening of multimorbidity and polyiatrogenesis. The case studies do not show that any specific treatment is irrational or inappropriate. What they show is that Greyfield patients receive a huge number of treatments and that these treatments have complex effects, many of them being bad. Due to the chronicity of both the problems and of the treatments, it seems impossible to avoid cascades of iatrogenic effects. At the same time, vertical systems do not allow doctors to control for iatrogenesis. When cascades of single-target treatments are administered without much explanation, the risk of polyiatrogenesis becomes even greater. Biomedical specificity becomes more dangerous, the more multimorbidity increases.

There are many similarities between Greyfielders and people in other deprived areas. For example, the cluster of “violence, immigration, depression, type 2 diabetes, and abuse” that Mendenhall (2012, 2016) found among Mexican immigrant women in the USA has significant overlaps with Greyfield syndemics. Violence, depression, diabetes, and abuse co-occur among Greyfielders as well. However, there are also significant differences. “Immigration” is not an important factor. Migration and ethnicity make a big difference in other areas of the UK, but not in Greyfield. Another key factor not present in Greyfield is a lack of healthcare access. Greyfield’s low life expectancy and high levels of premature multimorbidity cannot be attributed to any shortage of biomedical treatments. Greyfielders are socioeconomically deprived, but they do not lack access. In the NHS, treatments are free. There is absolutely no lack of targeted interventions. If anything, there is overtreatment. Many Greyfielders are multimorbid in their 30s and 40s not because they do not get treated, but *because* they get treated. Treatments have become part of the problem. GCC’s attempt to integrate complementary healing is a step in the right direction. LNC offers patients a space to reflect on their many mental, physical, and social problems. The therapists try to see patients not as carriers of distinct symptoms, but as whole people living complicated lives.

In the conjoint epidemics of multimorbidity and polyiatrogenesis, a more holistic approach to health is urgently needed. But complementary care cannot undo the harm done by deprivation and uncoordinated biomedical treatments, especially when it remains poorly integrated into the whole approach to health. LNC therapists could give food advice, but they were in no position to subtract any of the many medications that patients were on. They had no part in the biomedical management of cases. They were not even allowed access the GPs’ patient records. Their insights into multiple socioeconomic problems and multiple medications were not fed back to the doctors. How a truly complementary set of services could give “personalised, comprehensive continuity of care” (Barnett et al. 2012:37) to the people who suffer the most remains ill defined. As long as biomedicine splits mental, physical, and social health apart, whole health will remain elusive.
